# Aquaporin-2 in the early stages of the adenine-induced chronic kidney disease model

**DOI:** 10.1371/journal.pone.0314827

**Published:** 2025-01-30

**Authors:** Lucas H. Oronel, Maria Ortiz, Carolina Yarza, Sofía Gayone, Carlos Davio, Mónica Majowicz, Maria Florencia Albertoni Borghese

**Affiliations:** 1 Facultad de Farmacia y Bioquímica, Departamento de Ciencias Biológicas, Cátedra de Biología Celular y Molecular, Universidad de Buenos Aires, Buenos Aires, Argentina; 2 Facultad de Farmacia y Bioquímica, Departamento de Farmacología, Universidad de Buenos Aires, Buenos Aires, Argentina; 3 Consejo Nacional de Investigaciones Científicas y Técnicas (CONICET), Facultad de Farmacia y Bioquímica, Instituto de Investigaciones Farmacológicas (ININFA), Universidad de Buenos Aires, Buenos Aires, Argentina; 4 Consejo Nacional de Investigaciones Científicas y Técnicas (CONICET), Facultad de Farmacia y Bioquímica, Instituto de Química y fisicoquímica Biológicas (IQUIFIB), Universidad de Buenos Aires, Buenos Aires, Argentina; University of Pittsburgh, UNITED STATES OF AMERICA

## Abstract

Chronic kidney disease (CKD) is one of the leading health problems in the world. It is silent in the early stages and gradually progresses, inducing renal physiological and structural alterations. Moreover, CKD is associated with impaired life quality, increased risk for cardiovascular diseases, and reduced life expectancy. Different CKD animal models differ in underlying etiology, time of onset, and associated diseases. The 0.25% adenine diet induces progressive kidney damage, constituting an adequate model mimicking human CKD. Vasopressin (VP) was postulated as a mediator of CKD, mainly acting through its V2 receptors. However, the molecular mechanisms involved in the pathogenesis of this condition and its progression still are not entirely understood. This study aimed to evaluate if AQP2 expression is altered in an adenine-induced model of CKD in rats at early stages of development (two weeks) and to assess a potential beneficial effect of Tolvaptan (a V2 receptor antagonist) treatment. We showed an increased renal medullary AQP2 expression at two weeks of adenine administration. This increase was mainly cytoplasmic, explaining the increased urinary volume of CKD rats and suggesting a possible non-canonical role for AQP2. In addition, Tolvaptan effectively inhibited the V2 receptor in both control and CKD rats, decreasing AQP2 expression and increasing diuresis. Moreover, Tolvaptan slightly reduced BUN and plasma creatinine. On the other hand, the renal alterations induced by adenine in CKD rats were not prevented by Tolvaptan.

## Introduction

Chronic Kidney disease (CKD) is characterized by reduced kidney function (with a glomerular filtration rate, GFR, less than 60 ml/min per 1.73 m^2^) or the presence of kidney damage, sustained for a minimum of three months. Kidney damage could be detected through imaging studies or by kidney damage markers, such as hematuria or proteinuria [1].

CKD is one of the leading health problems in the world with a high global prevalence of 13.4% [2–4]. It is silent at early stages, progresses gradually, and can lead to kidney failure, which is its most severe form [2].

This disease is associated with impaired quality of life and reduced life expectancy at all ages increasing the risk for severe cardiovascular complications [2].

Although diabetes and hypertension are the main risk factors for CKD, there are other risk factors, such as glomerulonephritis, infections, polycystic kidney disease, and specific toxins. However, the molecular mechanisms involved in the pathogenesis and the progression of CKD are not completely understood [5].

A few years ago, plasma osmolarity, recurrent dehydration, and salt loss were demonstrated to drive CKD generation mechanisms [6]. Vasopressin (VP) was postulated as a mediator of CKD because acting through its V2 receptors promotes kidney disease progression by changing the composition of the tubular fluid at the macula densa, resulting in changes in the tubuloglomerular feedback control of GFR [7]. However, the mechanisms by which VP causes CKD are still under study except for the case of autosomal dominant polycystic kidney disease (ADPKD), a condition in which VP is known to play a role in cyst growth mediated by its second messenger cyclic adenosine monophosphate (cAMP) [7, 8].

Reducing VP secretion or blocking V2 receptors, either by increasing water intake or by using a V2 receptor antagonist, may benefit renal function in patients with all forms of CKD and those at risk of CKD [7, 8].

Studies done in 5/6 nephrectomy hypertensive rats have shown that increased water consumption, which decreases VP production, ameliorates the decline in GFR, histological damage, and proteinuria [9]. Another study in rats with renal mass reduction suggested VP participation in the development of CKD since a dual V1/V2 receptor antagonist slowed renal function deterioration [10].

Among all the possible mechanisms explaining VP involvement in CKD progression, one of the least studied was the VP role as a regulator of aquaporin-2 (AQP2). AQP2 is a water channel present in intracellular vesicles and in the apical membrane of principal cells of the collecting duct (CD) where it reabsorbs water and is responsible for the fine-tuning of urine osmolarity [11].

AQP2 can be regulated by VP-dependent and VP-independent mechanisms [12], although VP is the primary regulator of this water channel. VP, acting through its V2 receptor, regulates AQP2 expression and translocation to the apical membrane [13].

V2 receptor is a Gs-coupled receptor that binds VP and activates different adenylyl cyclases (AC), increasing intracellular cAMP levels. AC6 is the main isoform determining cAMP formation and AQP2 phosphorylation and trafficking in the principal cells of the renal medulla CD [14]. Exogenously added cAMP analogs replicate the rapid osmotic water permeability increase seen with VP, showing that the action of VP to increase water permeability in CD is mediated by cAMP [15].

One of the main effectors of cAMP is PKA, which is responsible for AQP2 phosphorylation at serine 256, a crucial event for AQP2 trafficking to the apical membrane [12]. There are additional phosphorylation sites in AQP2: serine 261, serine 264, and serine 269 (threonine 269 in humans), but the highest rate of phosphorylation was found in S256 which is considered a master switch for phosphorylation at other sites. Furthermore, phosphorylation at S256 is necessary for AQP2 trafficking from the Golgi to the apical membrane [16].

There is little information regarding AQPs and CKD. Decreased excretion of urinary AQP2 was found in humans with advanced CKD [17] and the function of the V2 receptor seems to be altered in advanced stages of CKD. However, to our knowledge, there is no information about AQP2 and the V2 receptor pathway at early stages of CKD [17].

Tolvaptan, a highly selective V2 receptor antagonist, has been approved for the treatment of ADPKD [18], volume management in congestive heart failure [19–21], and short-term fluid management after cardiac surgery [22]. A recent study has shown that Tolvaptan elicits renal protection in patients with deteriorated kidney function. However, the cellular and molecular mechanisms of this renal protection, particularly at early stages of CKD, remain unknown [22].

CKD animal models differ in underlying etiology, onset time, and CKD-associated diseases [23]. The 0.25% adenine diet induces continuous progressive kidney damage increasing plasma creatinine (PCr), blood urea nitrogen (BUN), plasma potassium concentration, proteinuria, and lactate dehydrogenase (LDH) activity, and decreasing clearance of creatinine and BUN [23]. The adenine-induced CKD has some advantages compared to other CKD animal models. In this model both kidneys remain available to perform determinations compared to the 5/6 nephrectomy model, it has low mortality and a slow induction of CKD. Besides, it allows the characterization of relatively stable kidney and cardiovascular disease, similar to CKD in humans [23].

Bearing in mind that AQP2’s role in CKD has not been fully established and that Tolvaptan has been shown to protect kidney function in advanced kidney disease but there are no studies at the early stages of kidney disease, this study aimed to evaluate if AQP2 expression is altered in an adenine-induced model of CKD in rats at early stages of CKD development (two weeks) and to assess a potential beneficial effect of Tolvaptan treatment.

## Materials and methods

### Animals and chronic kidney disease model

This study and all its protocols were reviewed and approved by the Institutional Animal Care and Use Committee (CICUAL) of the University of Buenos Aires, School of Pharmacy and Biochemistry under the protocol REDEC 2022–1439, and have been carried out following the guidelines of the International Council for Laboratory Science (ICLAS). Protocols followed the ARRIVE guidelines and included observing clinical, physiological, or behavioral signs that would lead to the application of a human endpoint. The experiments were conducted at the Department of Biological Sciences, School of Pharmacy and Biochemistry of the University of Buenos Aires. Research staff in animal care was trained and the researchers were granted the C category by the institutional committee, which allowed them to design and perform experiments with laboratory animals.

Male Sprague-Dawley rats aged 8–9 weeks were given 0.25% adenine in powdered standard rat food for two weeks to induce CKD (CKD group). Control animals of the same age received powdered standard rat food (C group). Another group received adenine and Tolvaptan (dried powder, Otsuka Pharmaceutical, Co., Ltd Tokyo, Japan) 30 mg/kg/day in the food concomitantly during the two weeks (CKD + T group) and the fourth group received only Tolvaptan (T group).

Body weight was registered before and after the adenine treatment. Animals were housed in a temperature and humidity-controlled environment with a 12 h light/dark cycle and were allowed free access to food and tap water. Wooden blocks were supplied to be gnawed and cardboard cylinders were added to enrich the environment. Animal health and behavior were monitored and scored daily, especially for general aspects, such as weight loss, lethargy, diarrhea, ruffled fur, or secretions. The human endpoint is immediately used if the score after the observations is ten or more or if there is anorexia or dehydration [24].

At the end of the adenine treatment period, animals were anesthetized with ketamine/xylazine (90 mg.Kg^-1^/10 mg.Kg^-1^), and blood samples were obtained by cardiac puncture until exsanguination [25].

### Determination of systolic blood pressure

Systolic blood pressure was recorded in all groups before and after the two-week adenine administration period in conscious rats by tail plethysmography (Sphygmomanometer Grass Medical Instruments, Model 7P8F, Quincy, Mass. USA).

### Plasma and urinary determinations

Twenty-four-hour urine samples were collected using metabolic cages at the end of the two-week period. Samples were analyzed for albumin by turbidimetric immunoassay, creatinine, urea, and urinary volume. Urinary albumin creatinine ratio (UACR) was calculated.

Plasma creatinine (PCr) and ureic nitrogen (BUN) were determined using kits provided by Wiener (Wiener Lab, Argentina). Urine osmolality was analyzed using a Wescor VAPRO osmometer model 5520 to measure the dew point temperature depression (Wescor Inc., Utah, USA).

### Tissue processing for histology and immunohistochemistry

Kidneys from control and treated rats were fixed in 4% phosphate buffer formaldehyde and embedded in paraffin. Kidney sections of 5 μm were processed for hematoxylin-eosin staining to analyze kidney structures´s morphology. Images from histological and immunohistochemical sections were captured using an Olympus 8J15816 light microscope coupled to a digital camera (Qcolor 3, Olympus America, Inc., Richmond Hill, Ontario, Canada).

### Immunohistochemistry for AQP2 and p256-AQP2

Five-micrometer slices were incubated with primary rabbit polyclonal antibody to AQP2 (Abcam Inc., Cambridge, MA, USA 1:500) or AQP2 phosphorylated in serine 256 (p256-AQP2) (Abcam Inc., Cambridge, MA, USA 1:200) and a secondary biotinylated donkey anti-rabbit (Jackson ImmunoResearch, West Grove, PA, USA, 1:750), followed by the streptavidin-biotin-peroxidase reaction (Dako Cytomation, Glostrup, Denmark) visualized by exposure to diaminobenzidine (DAB)-H_2_O_2_. Endogenous peroxidase activity was quenched with hydrogen peroxide to prevent unspecific staining. Tissue sections were counterstained with hematoxylin.

### Tissue processing for western blot analysis in medulla homogenates

Immediately after the animals were euthanized, their kidneys were isolated, and the renal medulla was dissected and homogenized in buffer (250 mmol/l sucrose, 1 mmol/l EDTA, 0.1 mmol/l PMSF and 10 mmol/l Tris-ClH), pH 7.6). Large tissue debris and nuclear fragments were removed by a low-speed spin (1000 g, 10 min, 4°C). Protein concentration was measured using a BCA TM Protein Assay Kit (Pierce, Rockford, IL, USA).

### Western blot for AQP2

Immunoblotting analysis was used to identify AQP2. AQP2 antibody (mouse monoclonal anti-rat AQP2; Santa Cruz Biotechnology, Inc.1:200) was followed by a peroxidase-conjugated secondary antibody donkey anti-mouse IgG (Jackson Immuno Research 1:5000) and revealed using SuperSignal West Pico Plus chemiluminescent substrate (Thermo Scientific, Rockford, IL, USA). Ponceau Red staining (0.2% in 5% Acetic acid) was used to assess protein loading. AQP2 antibody recognizes one band of 29 kDa corresponding to AQP2. The relative protein levels were analyzed with Image J 1.53e (NIH, USA), and the AQP2 to Ponceau Red ratio was calculated.

### Real-time PCR for TNF-alpha, interleukin-6, AQP2, V2 receptor and AC6

Total RNA was isolated using the SV total RNA Isolation System (Promega, Madison, WI, USA) and reverse transcribed to cDNA using a high-capacity reverse transcription kit (A&B, MA, USA). For real-time detection of TNF-alpha, interleukin-6 (IL-6), AQP2, V2 receptor, and AC6 transcripts and the reference gene (GAPDH), MezclaReal (Biodynamics, Buenos Aires, Argentina) and specific primers were used [26–28]. Primer sequences are reported in Table 1. The normalized gene expression method (2^–ΔΔCT^) was used for the relative quantification of gene expression [29].

**Table 1:** Sequences of the qPCR primers used.

Target gene	Forward primer (5´-3´)	Reverse primer (5´-3´)
*Gapdh*	GAAGGGCTCATGACCACAGT	GGATGCAGGGATGATGTTCT
*Aqp2*	CTGGTGCTGTGCATCTTTGC	ATGGAGCAACCGGTGAAA
*Avpr2*	CTCGGGCCTTCTCACTCCTT	CACTGCCATTTCCCACATCA
Adcy6	GGAGCACAACCCTTTGGCAT	GGCTGTTTTCCGTTCATCCAC
*Il6*	TTTCTCTCCGCAAGAGACTTC	TGGGTGGTATCCTCTGTGAA
*Tnf-alpha*	ATGGGCTCCCTCTCATCAGT	TCCGCTTGGTGGTTTGCTAC

### cAMP measurements

cAMP was measured in the renal medulla. Approximately 100 mg of renal medulla were homogenized in ice-cold absolute ethanol and centrifuged for 15 min at 1200 g. The supernatant was dried, and the remaining residue was resuspended for cAMP determination by competition of [3H]-cAMP for PKA [30]. Results were expressed as pmoles/mg of protein.

### Statistics

One-way ANOVA with Bonferroni’s post-test for multiple comparisons was performed using Graph Pad Prism version 5.0 for Windows.

The null hypothesis was rejected when p<0.05.

## Results

### Characterization of the animal model

Data for animal weight, renal functional parameters and systolic blood pressure (SBP) are shown in Table 2. After two weeks of adenine treatment, there were no significant differences in SBP.

**Table 2:** Summary of systemic and renal values and arterial blood pressure in control, CKD, CKD + T and T groups.

	Control	CKD	CKD + T	T
Initial SBP (mm Hg)	93.9 ± 0.4	97.5 ± 1.5	95.5 ± 2.5	90.3 ± 8.3
Final SBP (mm Hg)	89.5 ± 3.5	95.7 ± 5.3	101.8 ± 4.8	93.4 ± 4.3
Body weight change (%)	8.39 ± 2.32	0.71 ± 1.83*##	3.07 ±2.52	11.41 ± 1.27
Kidney weight (g)	1.509 ± 0.046	1.840 ± 0.058***###	1.756 ± 0.053*##	1.466 ± 0.047
Kidney weight / 100 g body weight (g/100g)	0.360 ± 0.008	0.467 ± 0.015***###	0.502 ± 0.023***###	0.383 ± 0.008
Blood Urea Nitrogen (BUN, mg/dL)	23.33 ± 2.80	36.87 ± 3.73 *##	31.27 ± 3.27	18.20 ± 0.47
Plasma creatinine (mg/L)	6.55 ± 0.28	9.44 ± 0.54*#	8.65 ± 0.59&	6.57 ± 0.38
Urinary volume (ml/24 h)	8.5 ± 1.4	20.1±3.2**	25.5 ± 2.2***##	14.8 ± 0.8 *
Albuminuria (mg/24h)	29.0 ± 14.5	78.3 ± 20.1*#	69.3 ± 26.1*#	29.4 ± 6.1
Urinary albumin creatinine ratio (UACR)	17.18 ± 3.92	38.62 ± 7.01*#	37.83 ± 2.89*#	20.22 ± 5.19
U Osmolality (mOsm/Kg H_2_O)	1285.0 ± 110.4	436.4 ±35.3***###	425.5 ± 32.4***###	971.3 ± 57.1

Each value represents the mean ± SEM of at least five independent determinations. ANOVA + Bonferroni *p < 0.05 vs. C; ** p<0.01 vs. C; ***p<0.005 vs. C; #p < 0.05vs T; ##p < 0.01 vs. T; ###p < 0.005 vs. T; & p < 0.05 vs. CKD. SBP: Systolic Blood Pressure.

Body weight increase was lower in CKD than in groups not receiving adenine. Kidney weight was significantly increased in both groups receiving adenine.

As expected, urinary volume increased in the adenine-induced CKD group due to renal malfunction and in Tolvaptan-treated rats. Urinary osmolality decreased in both CKD groups, treated and untreated with Tolvaptan, and in the control group treated with Tolvaptan. Between ten and fourteen animals per group were used for a total of 48 animals. No animals were found dead and there was no need to use a human endpoint as the animals were in a very good general condition.

### Two-week adenine administration increased blood urea nitrogen (BUN) and affected renal function

BUN and PCr were significantly higher in the CKD group than in the C and T groups demonstrating impaired renal function. When Tolvaptan was administered concomitantly with adenine, slightly attenuated PCr and BUN increases compared to the CKD group were observed, although this effect was only statistically significant for PCr.

Albuminuria was higher in both groups receiving adenine, and Tolvaptan did not affect this parameter. Similar results were obtained when the UACR was calculated.

### Water intake

**Fig 1:**
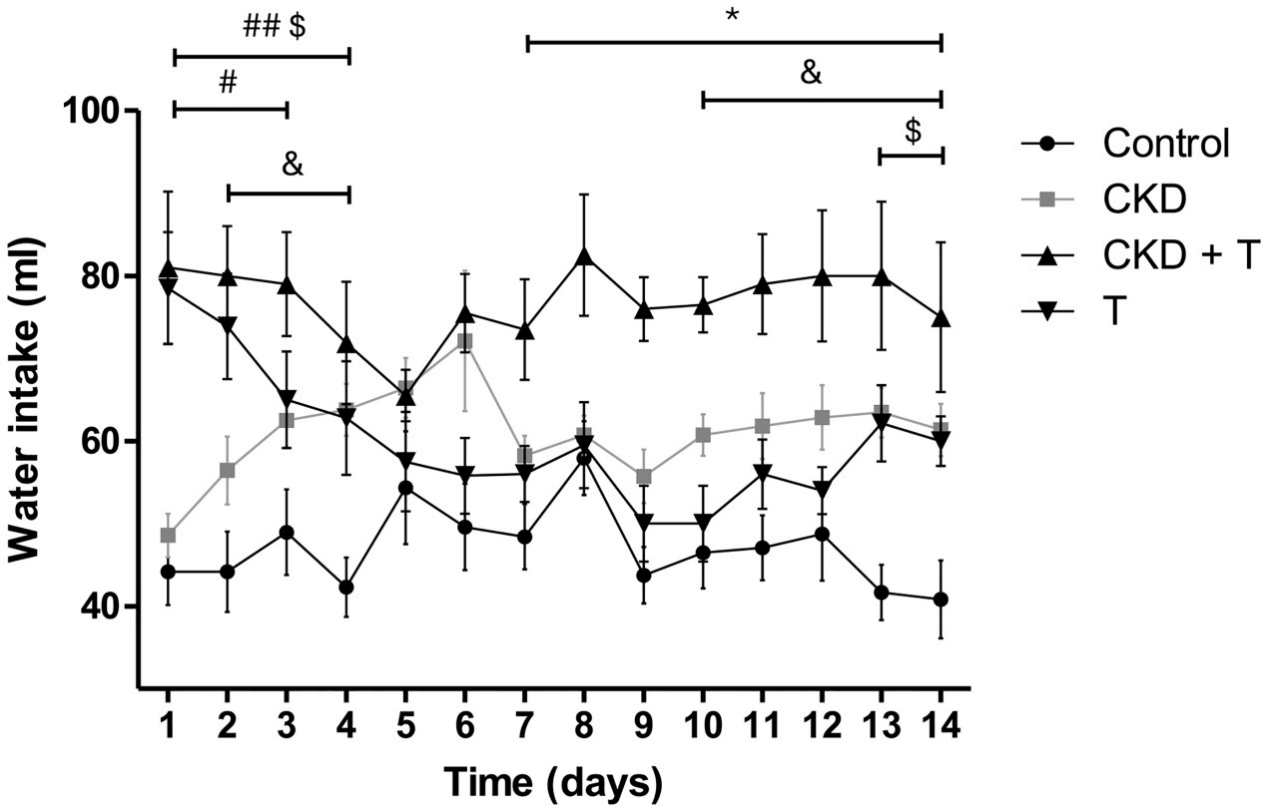
Daily water intake. Daily water intake from rats fed with control food (C group, black circle), 0.25% adenine in food (CKD group, gray square); 0.25% adenine + 30 mg/Kg/day Tolvaptan (CKD + T group, black triangle) or 30 mg/Kg/day Tolvaptan (T group, black inverted triangle). Each point represents the mean ± SEM of at least seven determinations. * p<0.05 CKD+T vs all other groups; # p<0.05 CKD+T vs. CKD; ## p<0.01 CKD+T vs. C; & p<0.05 C vs CKD; $ p<0.05 C vs. T.

CKD rats had significantly higher water consumption than control rats from days 2 to 4 and from day 10 to the end of the experiment. Both groups receiving Tolvaptan initially had higher water consumption than the untreated rats. After 3 days, control rats receiving Tolvaptan showed a decreased water consumption, having an intermediate value between control and CKD rats. On the other hand, the CKD + T group had significantly higher water consumption than the other three groups from day seven to the end of the experiment (Fig 1). This result suggests CKD rats are more sensitive to Tolvaptan than control rats. This result aligns with the urinary volume and urine osmolality determined at the end of the experiment.

### Two-week adenine administration affects the renal structure and increases inflammatory markers

**Fig 2:**
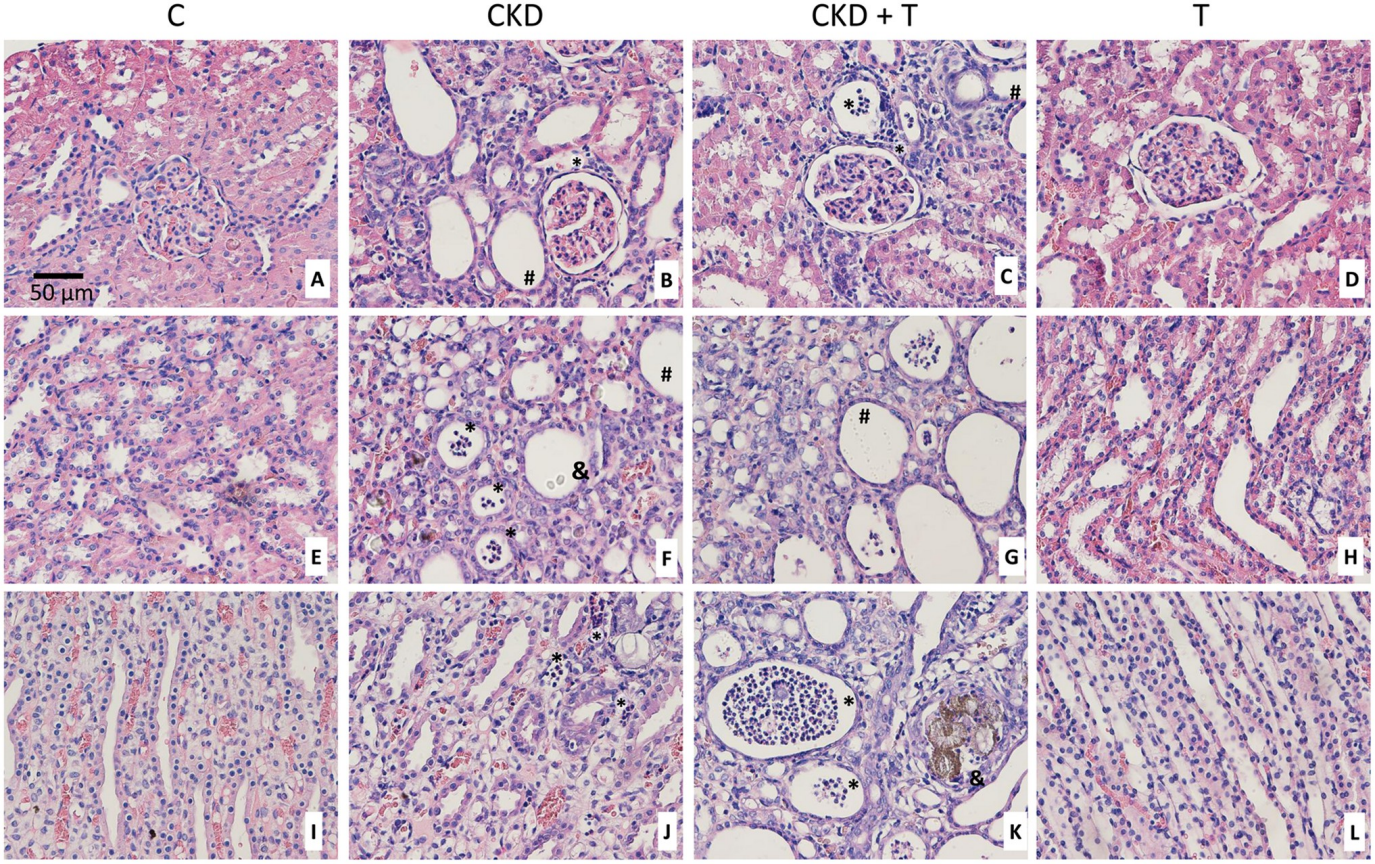
Histology of the kidney (A–L). Hematoxylin-Eosin-stained sections in kidneys from rats fed with control food (C group A, E, I), 0.25% adenine in food (CKD group B, F, J), 0.25% adenine + Tolvaptan 30 mg/Kg/day (CKD + T group C, G, K) or Tolvaptan (T group D, H, L) at a magnification of 400X. A-D Cortex, E-H Outer Medulla, I-L Inner Medulla. Inflammatory infiltration is marked as *, tubular dilatation as #, and crystal deposition as &. Representative images of at least four animals per group.

**Fig 3:**
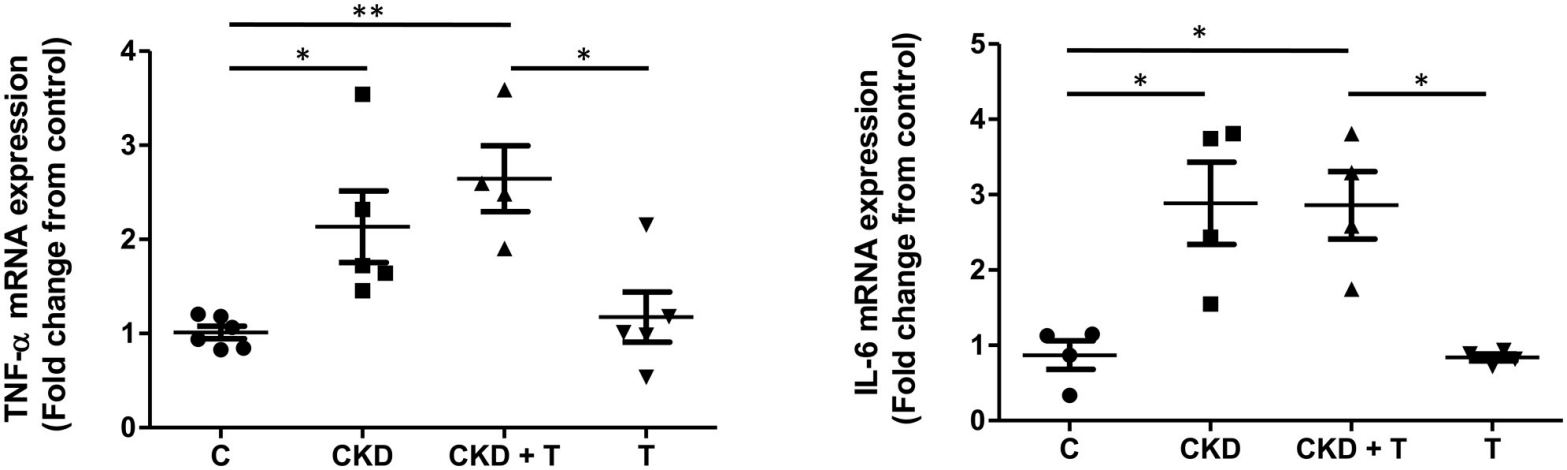
TNF-alpha and IL-6 mRNA expression in homogenates of the renal outer medulla. mRNA levels are expressed as relative values from control rats. C: Control, CKD: Adenine (0.25%), T: Tolvaptan (30 mg/Kg); CKD + T: Adenine (0.25%) + Tolvaptan (30 mg/Kg). Data are expressed as mean ± SEM of at least four independent experiments. *p<0.05 vs. indicated groups.

Adenine administration affected renal structure, as can be seen in Fig 2. After a two-week administration of adenine, we observed inflammatory cell infiltration, crystal deposition, flattening of the proximal tubule brush border, glomerular damage evidenced by sclerosis, and thickening of Bowman’s capsule. Inflammatory cytokines IL-6 and TNF-alpha mRNA were increased after the two-week adenine treatment (Fig 3). Concomitant administration of Tolvaptan did not prevent adenine-induced modifications in kidney structure or inflammatory markers (Figs 2 and 3).

### Two-week adenine administration alters aquaporin-2 expression in the outer medulla

**Fig 4:**
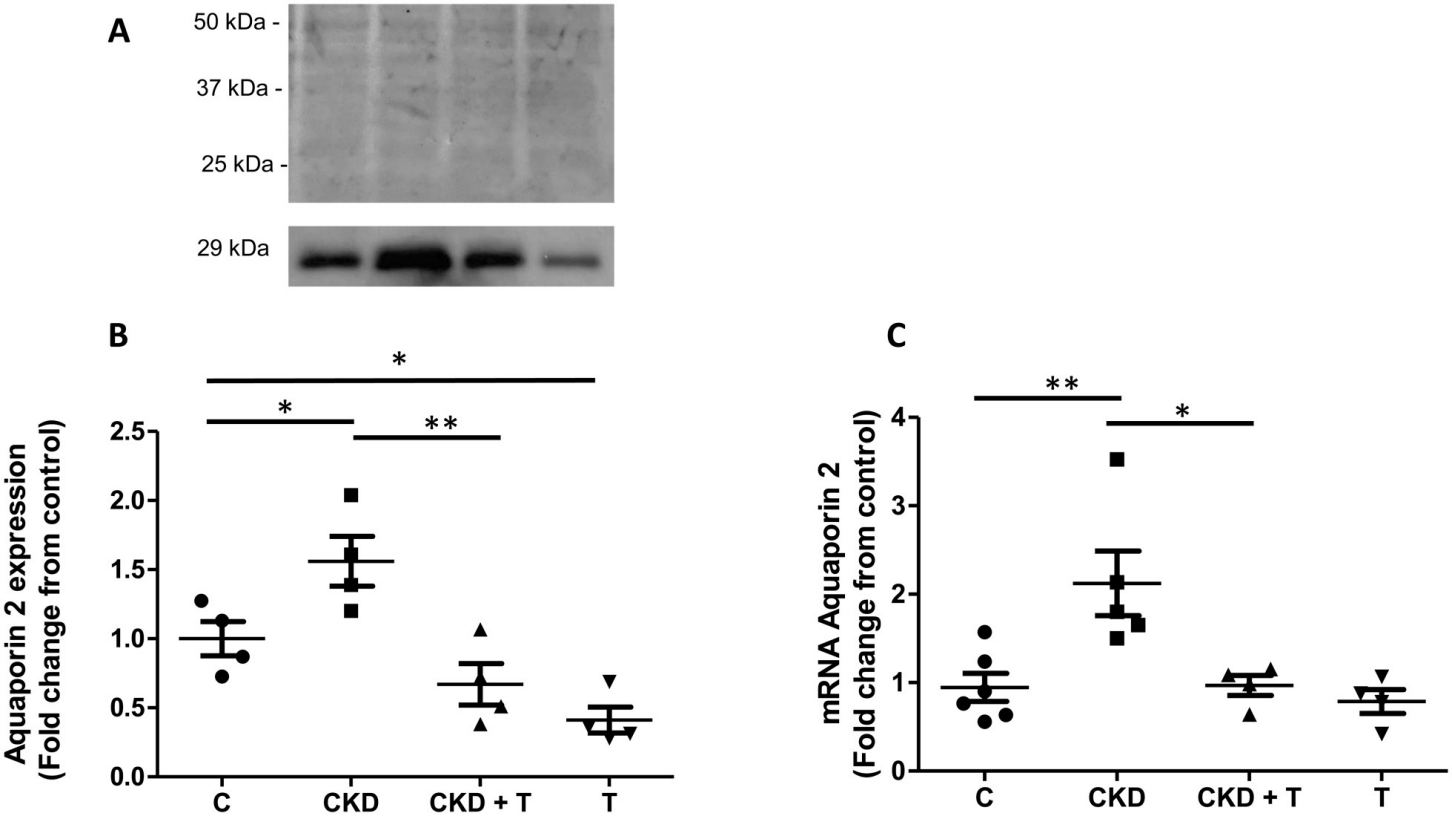
Aquaporin-2 protein and mRNA expression in homogenates of the renal outer medulla. **A** Representative blot showing Ponceau red staining of the blot and AQP2 (29 KDa) in control, CKD, CKD + T, and T rats. **B** Densitometric analysis of the 29 kDa AQP2 bands normalized by Ponceau red-stained bands. AQP2 protein expression is shown as AQP2 fold change from control rats. **C** AQP2 mRNA levels are expressed as relative values to control rats. C: Control, CKD: Adenine (0.25%), T: Tolvaptan (30 mg/Kg); CKD + T: Adenine (0.25%) + Tolvaptan (30 mg/Kg). Data are expressed as mean ± SEM of at least four independent experiments. *p<0.05 and **p<0.01 vs indicated groups.

AQP2 expression was significantly increased in adenine-treated animals (1.59 ± 0.20) compared to control animals (1.00 ± 0.11). Tolvaptan administration blocked this effect (A+T = 0.68 ± 0.15) as seen in Fig 4A and 4B. AQP2 expression in the group receiving Tolvaptan (T) decreased compared to the C group (0.42 ± 0.09 vs. 1.00 ± 0.11, respectively). AQP2 mRNA expression was increased in CKD (2.123 ± 0.366) compared to the C group (0.946 ± 0.159). This increase was prevented by Tolvaptan administration concomitant to adenine administration (0.968 ± 0.114). The T group had lower AQP2 mRNA values (0.787 ± 0.136) although not statistically significant versus the C group (Fig 4C).

### The increase in aquaporin-2 is due to a rise in cytoplasmic AQP2

**Fig 5:**
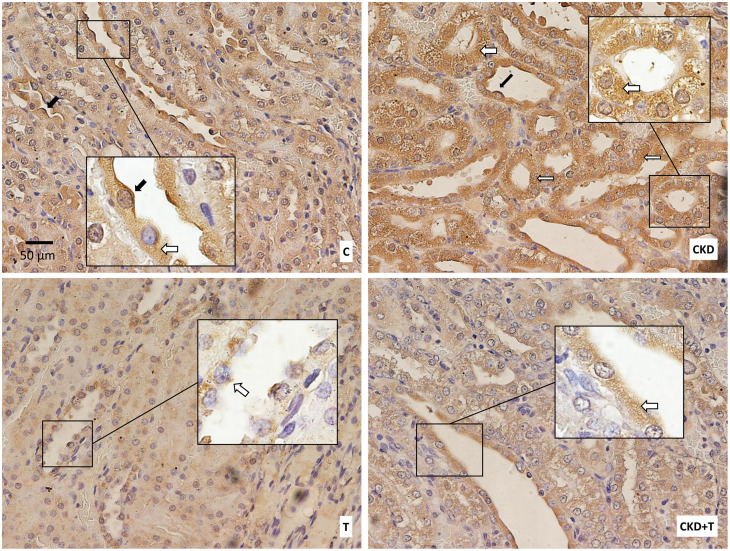
Aquaporin-2 protein expression in histological sections. Representative images of immunohistochemical staining for AQP2. All images have the same magnification: 400X. Insets show particular regions of the original images taken at higher magnification (1000x). Black arrows point to apical membrane AQP2; white arrows point to cytoplasmic AQP2. C: Control, CKD: Adenine (0.25%), T: Tolvaptan (30 mg/Kg); CKD + T: Adenine (0.25%) + Tolvaptan (30 mg/Kg). Images are representative of at least four animals per group.

To corroborate the results obtained by western blot, we performed an immunohistochemical assay for AQP2. The immunohistochemistry showed similar results to the western blot: AQP2 labeling appeared to increase in the CKD group at the CD level compared to the control group, and this increase was prevented by Tolvaptan (CKD + T group). As expected, AQP2 labeling in control rats receiving Tolvaptan (T group) was weaker than in control rats (C group). Another interesting result was that AQP2 labeling was mainly located in the cytosol of CD cells in the CKD group. In contrast, it was located in the cytosol and the apical plasma membrane in CD cells of control rats (Fig 5).

**Fig 6:**
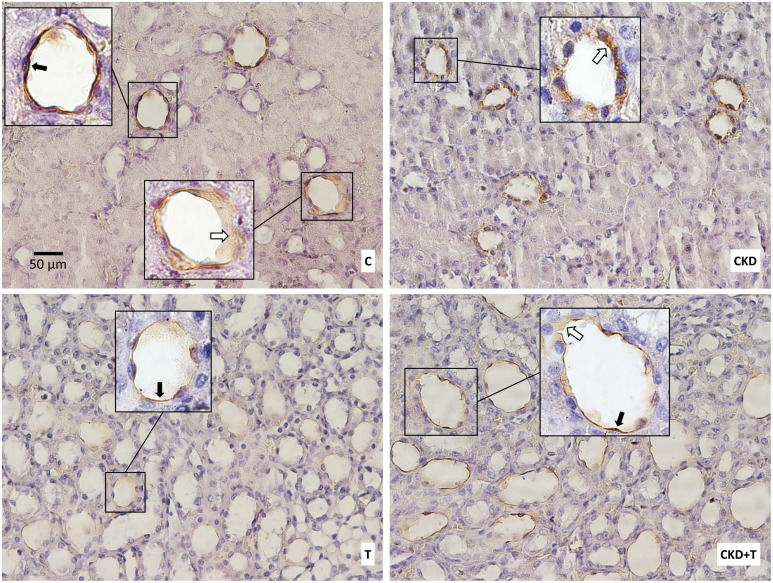
Aquaporin-2 phosphorylated in serine 256 (p256-AQP2) expression in histological sections. Representative images of immunohistochemical staining for p256-AQP2. All images have the same magnification: 400X. Insets show particular regions of the original images taken at higher magnification (1000x). Black arrows point to apical membrane AQP2; white arrows point to cytoplasmic AQP2. C: Control, CKD: Adenine (0.25%), T: Tolvaptan (30 mg/Kg); CKD + T: Adenine (0.25%) + Tolvaptan (30 mg/Kg). Images are representative of at least four animals per group.

To have a more specific assay, we performed immunohistochemistry for p256-AQP2 and we also found an increase in p256-AQP2 in the CKD group compared to the control group. The pattern of expression of p256-AQP2 was different among groups. In the CKD group, the signal was primarily detected in the cytoplasm showing a difference from the control group in which the signal was located mainly in the apical membrane and in the cytoplasm. p256-AQP2 was markedly decreased in both groups receiving Tolvaptan (CKD + T and T), being barely detectable in the T group, where p256-AQP2 was less expressed than in the C group and CKD+T group (Fig 6).

### Two-week adenine administration alters cAMP production and adenylyl cyclase six expression without changes in V2 receptor expression in the outer medulla

**Fig 7:**
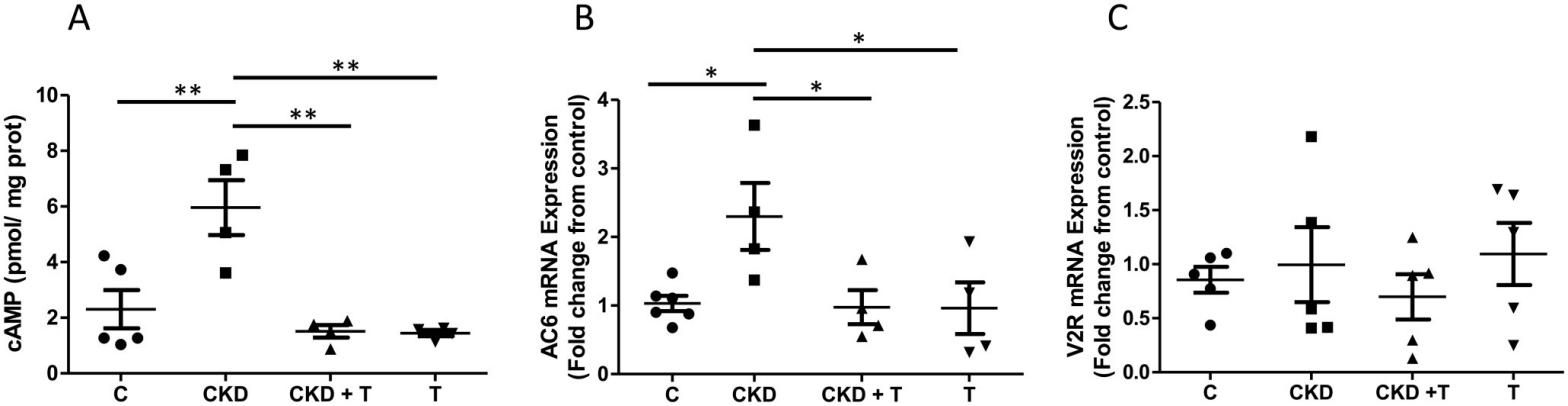
cAMP concentration, adenylyl cyclase 6 expression, and V2 receptor expression in renal medulla. A: cAMP concentration assessed by competition of [3H]-cAMP for PKA. B: Adenylyl cyclase 6 mRNA expression levels are expressed as relative values from control rats. C: V2 receptor mRNA levels are expressed as relative values from control rats. One-way ANOVA followed by Bonferroni´s test, *p<0.05; **p<0.01 vs. indicated groups. C: Control, CKD: Adenine (0.25%), T: Tolvaptan (30 mg/Kg); CKD + T: Adenine (0.25%) + Tolvaptan (30 mg/Kg). Data are shown as mean ± SEM of at least four independent experiments.

cAMP production increased in the CKD group compared to the C group (C: 2.230 ± 0.751 vs CKD: 5.960 ± 0.989 pmol/ mg protein), and this result was prevented by concomitant administration of Tolvaptan (CKD + T: 1.510 ± 0.070 pmol/mg protein; T: 1.320 ± 0.44 pmol/ mg protein); these results are shown in Fig 7A. AC6 mRNA expression showed the same pattern, as seen in Fig 7B (fold change from control; C: 1.03 ± 0.11; CKD: 2.30 ± 0.49; CKD + T: 0.98 ± 0.35; T: 0.96 ± 0.38), while V2 receptor mRNA expression was not significantly modified by CKD or Tolvaptan administration (Fig 7C fold change from control; C: 0.85 ± 0.20; CKD: 0.90 ± 0.43; CKD + T: 0.65 ± 0.26; T: 1.05 ± 0.37).

## Discussion

Based on VP’s contribution to diabetic and non-diabetic CKD progression [31], VP’s role as the primary regulator of AQP2 through the V2 receptor, and increased diuresis in CKD animal models [8], we studied the VP axis and AQP2 expression and localization early in the disease.

Our results show that 0.25% adenine administration in powdered food constitutes an acceptable model of CKD, since BUN, PCr, diuresis, UACR, and albuminuria significantly increased after two weeks of adenine administration (Table 2). Tolvaptan treatment improved kidney function as shown by a decrease in PCr levels. We also observed the structural renal alterations previously described by other authors in adenine-fed rats at this early time point of the disease. At the tubular level, we found atrophy, proximal tubular brush border erosion, and flattening of the epithelium. We also found glomerulosclerosis and inflammatory infiltration, as described by Diwan et al. (Fig 2) [23]. Inflammatory markers TNF-alpha and IL-6 were increased in the CKD group (Fig 3) as previously reported in the same model at a later time point [32, 33]. These renal alterations were not prevented by Tolvaptan administration.

We did not find changes in SBP after two weeks of adenine administration. On the other hand, Tolvaptan administration did not modify SBP levels in control nor in CKD rats (Table 2). In contrast with our results, Diwan et al. reported increased SBP after a 0.25% adenine diet in rats, after four weeks of adenine administration [34]. Fong et al. found a significant increase in BP after two weeks of adenine administration, but in this case, the dose of adenine was higher than ours and was administered by oral gavage [35]. We also found that kidney weight was significantly higher in CKD rats than in control animals, despite their lower body weight (Table 2). These results are similar to other reports showing both kidneys in CKD animals were hypertrophied [23]. Tolvaptan administration did not modify this hypertrophic response.

Furthermore, AQP2 expression and localization were altered in this animal model (Figs 5 and 6). Contrary to our expectation, we found that AQP2 expression was increased in the outer medulla of adenine-induced CKD rats, despite increased urinary volume and decreased urinary osmolality. We also detected a significant increase in renal medullary cAMP levels and AC6 expression in CKD rats compared to control rats, which aligns with the results of AQP2 expression (Fig 7).

To our knowledge, only a few studies evaluated AQP2 in adenine-induced CKD with opposite results to ours, i.e., decreased AQP2 expression, but in all these cases there were differences in the dose of adenine, the experimental time, or the species evaluated. For example, Dos Santos et al. reported a decreased renal AQP2 protein expression in rats treated with a 0.5% adenine diet [36]. Recent work from Atay et al. reported a decreased inner medullary AQP2 expression and translocation to the plasma membrane in the adenine-CKD model. Still, it was performed in mice for a more prolonged adenine administration (4 weeks) [37]. These adenine-treated mice had impaired renal function, accompanied by tubular injury, inflammation, and fibrosis at the end of adenine treatment. Interestingly, the authors found a recovery of most renal functional parameters, with increased AQP2 membrane targeting after a two-week recovery period. However, they only found partial recovery of tubular injury and inflammation [37].

Kakeshita et al. found a decrease in urinary cAMP and AQP2 in patients with CKD despite increased VP levels. They explained that in patients with advanced CKD, the CD cannot respond to the stimulation of VP [17].

Teitelbaum et al. had previously reported that VP resistance was due to mRNA V2 receptor downregulation in the 5/6 nephrectomy CKD model [38]. However, in contrast with these studies, we did not find changes in renal medullary V2 receptor mRNA in two-week adenine-induced CKD rats.

Another study by Oshikawa-Hori et al., performed in healthy volunteers (controls) and CKD patients, evaluated AQP1 and AQP2 in urinary extracellular vesicles (uECVs). In this study, the CKD patients were classified into different groups according to the severity of CKD and the results obtained suggested that both AQP1 and AQP2 were decreased in uECVs from patients with advanced CKD and could serve as new biomarkers for the diagnoses of advanced CKD [39]. Again, decreased levels of AQP2 are found in advanced CKD compared to our results at an earlier time-point of the disease.

Interestingly, Cano Peñalver et al have shown that Integrin-linked kinase (ILK) regulates AQP2 expression and localization independently of VP and that AQP2 and ILK colocalize in cultured inner medullary collecting duct (mIMCD3) cells [40]. ILK contributes to the organization of the dynamic cytoskeletal architecture during AQP2 recycling, a crucial event for its exocytotic pathway [41]. The same group demonstrated in the adenine-induced mouse model of CKD a progressive increase in ILK expression and activity in the kidney [42]. However, they did not explore AQP2 expression in these mice.

The increased diuresis with increased AQP2 expression in our experimental model can be explained by the fact that AQP2 localization in adenine-induced CKD rats is mainly cytoplasmic and not in the apical membrane where it should be to reabsorb water (Fig 5). On the other hand, in control rats, AQP2 is localized in the cytoplasm and the apical plasma membrane of the principal cells of the CD (Fig 5). p256-AQP2 showed the same pattern as total AQP2, which increased in the CKD group, with cytoplasmic localization explaining the increased urinary volume (Fig 6). As expected, Tolvaptan administration markedly decreased AQP2 and p256-AQP2 expression in both control and CKD rats (Figs 4–6).

One possible explanation for the cytoplasmic increase in AQP2 in CKD rats could be impaired protein trafficking to the plasma membrane. It is known that cAMP has several effector proteins, such as PKA, EPAC, cyclic nucleotide-gated channels, and Popeye domain-containing proteins (POPDC) [43]. Recently it has been reported that POPDC proteins are expressed in diverse organs, including the kidney, and exert different functions, such as cell-cell contact formation and control of vesicular transport and fusion [44]. In MDCK cells, a renal epithelial cell line, it was shown that POPDC1 (also called BVES: blood vessel epicardial substance) colocalizes with VAMP3, an essential protein for vesicular trafficking [45]. Considering this, changes in POPDC1 could alter AQP2 trafficking regardless of increased cAMP.

Furthermore, compartmentalized cAMP signaling is crucial for AQP2 trafficking to the plasma membrane and the interaction of PKA with specific AKAPs (A-kinase anchoring proteins) is a prerequisite for this AQP2 translocation. At least four AKAPS are involved in AQP2 trafficking regulation [5]. The altered expression or activity of those specific AKAPs would impair AQP2 translocation to the plasma membrane and might explain AQP2 cytoplasmic localization in this CKD model.

AQP2 exocytosis requires an active cytoskeleton network. Liu et al. showed that Actin-related protein 2/3 (Arp2/3), a protein involved in actin polymerization that colocalizes with AQP2, is crucial for AQP2 exocytosis [46]. Considering this, altered AQP2 trafficking to the plasma membrane may also be the consequence of an impaired cytoskeleton network.

Another possibility is that the cytoplasmic AQP2 pool is ready to exert other functions than water transport. Although the central or conventional known role of AQP2 is to transport water, non-canonical roles have been described, such as its contribution to cell migration, proliferation, differentiation, or apoptosis [47–51].

## Conclusion

Our findings show that AQP2 expression is increased in the renal medulla early in the development of the 0.25% adenine-induced CKD model. This increase in AQP2 expression is mainly cytoplasmic which accounts for the increased urinary volume of CKD rats. The cytoplasmic increase in AQP2 in CKD rats might be explained by impaired protein trafficking to the plasma membrane. Alternatively, cytoplasmic AQP2 could exert other non-canonical roles than water transport, i.e. contributing to cell migration, proliferation, differentiation, or apoptosis.

In addition, Tolvaptan effectively inhibited V2 receptors in both control and CKD rats decreasing AQP2 expression and increasing diuresis. Moreover, Tolvaptan slightly reduced BUN and plasma creatinine at this early experimental time of CKD development, although BUN changes were not statistically significant. On the other hand, the renal morphological alterations and the increase in inflammatory markers induced by adenine in CKD rats were not prevented by Tolvaptan. However, it was administered only for two weeks, and perhaps more time is needed to evaluate its effect on renal morphological alterations.

The efficacy of Tolvaptan in CKD is still under evaluation and depends on the origin and associated comorbidities of CKD, as well as the disease stage. In addition, we have to consider that there are responders and non-responders to Tolvaptan [52]. Some studies reported a beneficial effect of Tolvaptan in combination with other drugs. For example, the Tolvaptan/Valsartan combination improves cardiac and renal functions and prevents the fibrosis, inflammation, and apoptosis of heart and kidney from Doxycycline-treated mice [53].

In conclusion, the adenine-induced CKD model is a valuable tool for assessing the molecular mechanisms of the disease and therapeutic strategies in preclinical CKD studies [54]. Furthermore, our study suggests that V2 inhibition could be considered an adjunct therapy to slow the progression of CKD. Therefore, this promising research opens up new possibilities for the future treatment of CKD.

## Supporting information

S1 Fig Original and uncropped blots. (TIF)
