# Supplementary figures and images for: Aquaporin-2 in the early stages of the adenine-induced chronic kidney disease model
Source: PLoS One. 2025 Jan 30;20(1):e0314827. doi: 10.1371/journal.pone.0314827 (PMC11781631; doi:10.1371/journal.pone.0314827)

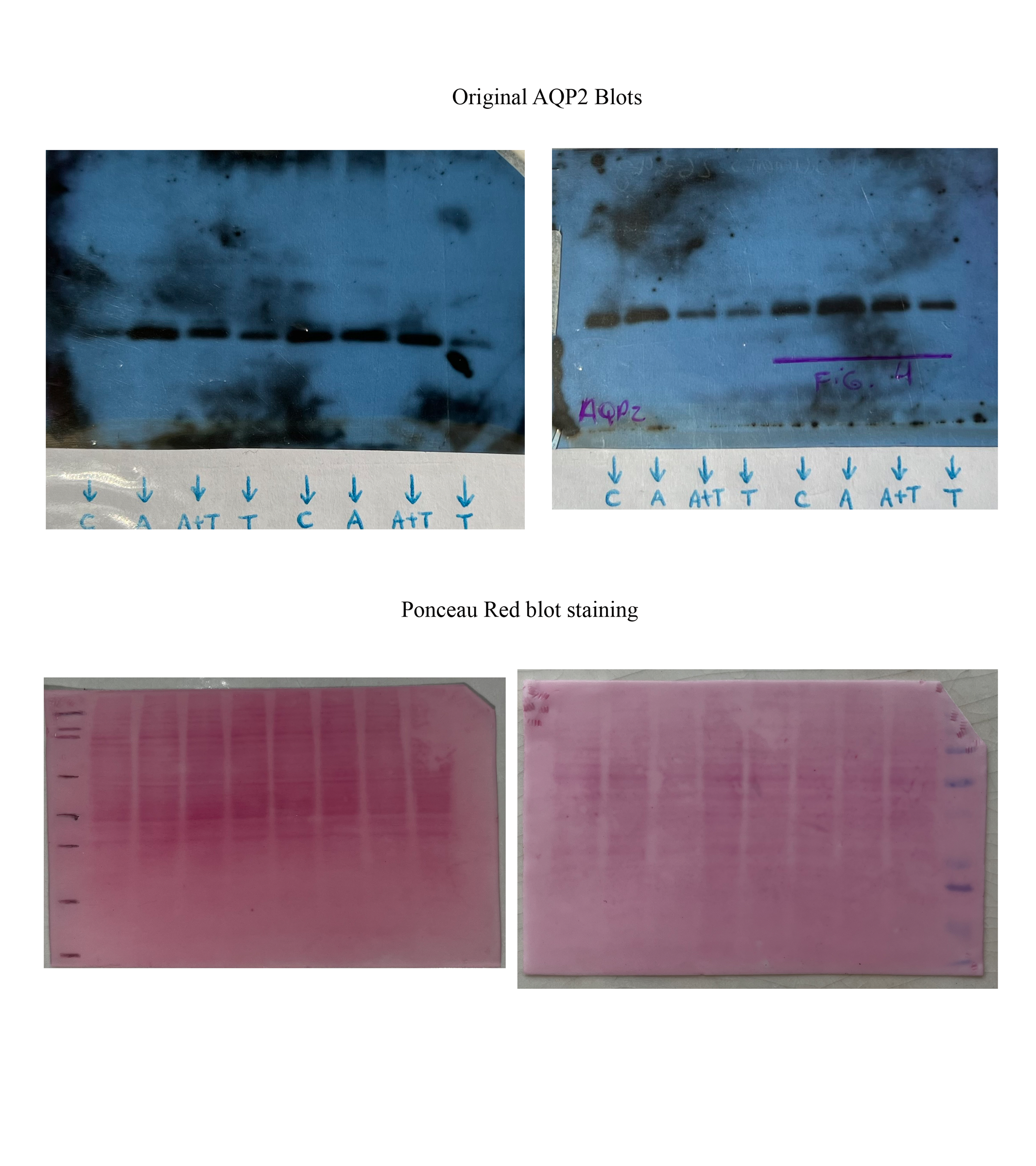

Supplement: S1 Fig — (TIF) [file pone.0314827.s001.tif]
